# Comparison of Cancer Prevalence in Patients With Neurofibromatosis Type 1 at an Academic Cancer Center vs in the General Population From 1985 to 2020

**DOI:** 10.1001/jamanetworkopen.2021.0945

**Published:** 2021-03-18

**Authors:** Jace P. Landry, Kelsey L. Schertz, Yi-Ju Chiang, Angela D. Bhalla, Min Yi, Emily Z. Keung, Christopher P. Scally, Barry W. Feig, Kelly K. Hunt, Christina L. Roland, Ashleigh Guadagnolo, Andrew J. Bishop, Alexander J. Lazar, John M. Slopis, Ian E. McCutcheon, Keila E. Torres

**Affiliations:** 1 Department of Surgical Oncology, University of Texas MD Anderson Cancer Center, Houston; 2 Department of Breast Surgical Oncology, University of Texas MD Anderson Cancer Center, Houston; 3 Department of Radiation Oncology, University of Texas MD Anderson Cancer Center, Houston; 4 Department of Pathology, University of Texas MD Anderson Cancer Center, Houston; 5 Department of Neuro-Oncology, University of Texas MD Anderson Cancer Center, Houston; 6 Department of Neurosurgery, University of Texas MD Anderson Cancer Center, Houston

## Abstract

**Importance:**

Neurofibromatosis type 1 (NF1) is a complex genetic disorder that is associated with not only neurofibromas, but also an increased susceptibility to other neoplasms.

**Objective:**

To evaluate the prevalence of neoplasia and outcomes among patients with NF1.

**Design, Setting, and Participants:**

This cohort study was conducted among patients with NF1 at a single academic cancer center from 1985 to 2020 with median (range) follow-up of 2.9 years (36 days to 30.5 years). Of 2427 patients evaluated for NF1, 1607 patients who met the National Institutes of Health consensus criteria for NF1 were included. This group was compared with estimates from Surveillance, Epidemiology, and End Results (SEER) Cancer Statistics Review 1975 to 2015 and SEER participants database unless otherwise specified. Data were analyzed from August 2018 to March 2020.

**Main Outcomes and Measures:**

Disease-specific survival (DSS) was measured from diagnosis date to date of neoplasm-specific death or censorship and calculated using the Kaplan-Meier method. Survival curves were compared using the log-rank test. Deaths from disease were considered a DSS end point; other deaths were considered censored observations. Secondary outcome measures were comparisons of (1) overall survival of patients with NF1 with neurofibroma neoplasms vs those without nonneurofibroma neoplasms, (2) neoplasm prevalence in the NF1 group vs general population estimates, and (3) age at diagnosis in the NF1 group vs general population estimates for the most common neoplasms in the NF1 group.

**Results:**

Among 1607 patients with NF1, the median (range) age at initial visit was 19 years (1 month to 83 years) and 840 (52.3%) were female patients. Among 666 patients who developed other neoplasms in addition to neurofibromas (41.4%), 295 patients (18.4%) developed glioma and 243 patients (15.1%) developed malignant peripheral nerve sheath tumor (MPNST), the most common neoplasms. Patients with NF1, compared with the general population, developed several neoplasms at a younger mean (SD) age (low-grade glioma: 12.98 [11.09] years vs 37.76 [24.53] years; *P* < .0001; high-grade glioma [HGG]: 27.31 [15.59] years vs 58.42 [19.09] years; *P* < .0001; MPNST: 33.88 [14.80] years vs 47.06 [20.76] years; *P* < .0001; breast cancer: 46.61 [9.94] years vs 61.71 [13.85] years; *P* < .0001). Patients with NF1 developed neoplasms more frequently compared with the general population (odds ratio, 9.5; 95% CI, 8.5-10.5; *P* < .0001). Among patients with NF1, significantly lower 5-year DSS rates were found among those with undifferentiated pleomorphic sarcoma (1 of 5 patients [20.0%]), HGG (8 of 34 patients [23.1%]), MPNST (72 of 228 patients [31.6%]), ovarian carcinoma (4 of 7 patients [57.1%]), and melanoma (8 of 12 patients [66.7%]) compared with those who had neoplasms classified as *other* (110 of 119 patients [92.4%]) (all *P* < .001) .

**Conclusions and Relevance:**

This cohort study found that among patients with NF1, those who developed undifferentiated pleomorphic sarcoma, HGG, MPNST, ovarian carcinoma, or melanoma had significantly lower DSS rates compared with those who developed other neoplasms. This study also found that patients with NF1 developed some neoplasms more frequently and at a younger age compared with individuals without NF1. HGGs and MPNST were noteworthy causes of death among patients NF1. This information may be useful for NF1 patient counseling and follow-up.

## Key Points

**Question:**

Do patients with neurofibromatosis type 1 (NF1) develop neoplasms differently or experience worse outcomes compared with the general population?

**Findings:**

In this cohort study of 1607 patients with NF1 evaluated at an academic cancer center who developed neoplasms other than neurofibromas, patients developed neoplasms at a younger age and more frequently compared with general population estimates. Patients with undifferentiated pleomorphic sarcoma, high-grade glioma, malignant peripheral nerve sheath tumor, ovarian carcinoma, and melanoma had significantly lower disease-specific survival rates compared with patients with other neoplasms.

**Meaning:**

These findings suggest that patients with NF1 develop several neoplasms, including gliomas and sarcomas, at a younger age, more frequently, and with worse outcomes compared with individuals without NF1 and that counseling and annual follow-up should be advised for these patients.

## Teaser

This cohort study examines the cancer prevalence and survival outcomes among patients with neurofibromatosis type 1 vs the general population.

## Introduction

Neurofibromatosis type 1 (NF1) is an inherited autosomal-dominant disorder that occurs in 1 in 3000 individuals.^1^ Manifestations of NF1 can be associated with outcomes in every organ system.^2^ Patients with NF1 have wide phenotypic variability, and the condition is associated with higher rates of benign and malignant tumors.^3,4,5^ Life expectancy is 10 years to 15 years shorter than that among the general population, a decrease associated with malignant neoplasms.^6^ Although an association with certain neoplasms in NF1 is well-recognized, the risk of specific neoplasms has been challenging to quantify accurately.^3^ This study aimed to determine the prevalence, management, and outcomes among patients with NF1 who developed neoplasms other than neurofibromas and received treatment at a large multidisciplinary cancer center.

## Methods

The University of Texas MD Anderson Cancer Center Institutional Review Board (IRB) approved this cohort study. The IRB granted a waiver of informed consent because the study is a retrospective medical record review with no more than minimal risk and the collected, limited data were double coded, so the link is known only to researchers. Our study adhered to the Strengthening the Reporting of Observational Studies in Epidemiology (STROBE) reporting guideline with a completed checklist for observational cohort studies in epidemiology.

### Study Population

**Figure 1.**
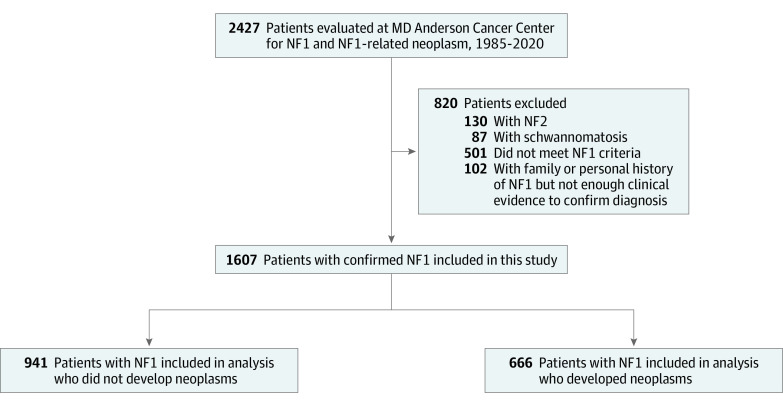
Flow Chart for Inclusion and Exclusion Criteria NF1 indicates neurofibromatosis type 1; NF2, neurofibromatosis type 2.

We identified patients evaluated for NF1 or an NF1-related neoplasm from 1985 to 2020 (Figure 1; Table 1). An unblinded electronic health record review was performed by 2 research fellows (J.P.L. and K.L.S.). Patients identified with an *International Statistical Classification of Diseases and Related Health Problems, Tenth Revision* (*ICD-10*)^7^ diagnosis code of neurofibromatosis or an NF1-defining lesion, such as malignant peripheral nerve sheath tumor (MPNST), optic pathway glioma, or neurofibromas, were included. Patients were included if they had sufficient documentation supporting NF1 clinical diagnosis according to the updated 1987 National Institutes of Health consensus criteria (eTable 1 in the Supplement).^8,9^ Patients with incomplete data were excluded. Patient data were anonymized prior to analysis, and data abstraction was agreed upon prior to collection and reviewed during collection by the research team. In specific cases in which the 2 research fellows did not agree on the criteria of inclusion, a third blinded research expert (I.E.M.) was involved.

**Table 1.** Patient Characteristics

Characteristic	Patients, No. (%) (N = 1607)
Age at initial visit, median (range), y	19 (1 mo-83 y)
Sex	
Male	767 (47.7)
Female	840 (52.3)
Race/ethnicity	
Caucasian	974 (60.6)
Hispanic	305 (19.0)
African American	253 (15.7)
Other^a^	75 (4.7)
Family history of NF1	
Yes	629 (39.1)
No	970 (60.4)
Not reported or unknown	8 (0.5)
NF1 genetic testing	
Known or reported	271 (16.9)
No testing or unknown genetic status	1336 (83.1)
Cutaneous neurofibroma	
Present	1163 (72.4)
Absent	442 (27.5)
Unknown or not evaluated	2 (0.1)
Café au lait macules	
Present	1386 (86.2)
Absent	26 (1.6)
Unknown or not evaluated	195 (12.1)
Skin fold freckling	
Present	817 (50.8)
Absent	322 (20.0)
Unknown or not evaluated	468 (29.1)
Lisch nodules	
Present	483 (30.1)
Absent	461 (28.7)
Unknown or not evaluated	663 (41.3)
Deep focal or plexiform neurofibroma	
Present	894 (55.6)
Absent	713 (44.4)

Abbreviation: NF1, neurofibromatosis type 1.

^a^ Includes 43 Asian Americans, 30 Arab Americans, and 2 Native Americans.

The primary outcome measure was disease-specific survival (DSS). Secondary outcome measures were comparisons of (1) overall survival of patients with NF1 with nonneurofibroma neoplasms vs those without nonneurofibroma neoplasms, (2) neoplasm prevalence in the NF1 group vs general population estimates, and (3) age at diagnosis in the NF1 group vs general population estimates for the most common neoplasms.

The date of neoplasm diagnosis was the date of histological confirmation following expert pathological evaluation. For patients not undergoing resection, the diagnosis date was the earliest date at which the neoplasm was noted on imaging.^10^ General population estimations for neoplasm prevalence, median diagnosis age, and 5-year DSS were obtained from the Surveillance, Epidemiology, and End Results (SEER) Cancer Statistics Review 1975 to 2015; the SEER participants database (1985-2017); and several large studies.^3,11,12,13,14,15,16,17,18,19,20,21,22^ Type of treatment received for specific neoplasms, including surgical treatment, chemotherapy, and radiation therapy, were recorded. Surgical treatment was defined as surgical resection of the specific neoplasm, chemotherapy was defined as any systemic agent used to treat the specific neoplasm, and radiation therapy was defined as neoplasm-specific treatment using forms of radiation.

### Statistical Analysis

Comparisons of neoplasm prevalence in the NF1 group with the general population were calculated using SEER prevalence data and large population databases referenced in Table 2. Hypothesis tests were 2-tailed analyses. The odds ratios (ORs) and 95% CIs of specific neoplasms in patients with NF1 compared with the general population were calculated using the χ^2^ test with Yate correction and Woolf logit method (Table 2). To compare mean age at diagnosis for specific neoplasms, we used SEER*Stat statistical software version 8.3.5 (US Department of Health and Human Services) to query data from SEER registries for patients diagnosed with glioma, glioblastoma, anaplastic astrocytoma, pilocytic astrocytoma, anaplastic oligodendroglioma, ependymoma, astrocytoma, breast cancer, or MPNST from 1985 through 2017. Two-sample Wilcoxon rank-sum test and t test were used to compare the mean age at diagnosis of both groups.

**Table 2.** Prevalence, Age at Diagnosis, and Disease-Specific Survival of Patients With Nonneurofibroma Neoplasms

Neoplasm type	NF1 group (N = 1607)			General population estimates^a^			OR (95% CI)
	Prevalence, No. (%)	Age at diagnosis, median (range), y^b^	5-y DSS, No. (%)	Prevalence, %	Age at diagnosis, median (range), y^b^	5-y DSS, No. (%)	
Nonneurofibroma neoplasms	666 (41.4)	NA	NA	NA	NA	NA	NA
Single neoplasm	550 (34.2)	NA	NA	5.5^22^	NA	NA	9.5 (8.5-10.5)^c^
Multiple neoplasms	116 (7.2)	NA	NA	NA	NA	NA	NA
Glioma							
Low grade	267 (16.6)	11.0 (0.1-56.8)	118 (98.1)	0.003	9.0 (0-19.0)	4040 (92.0-94.0)^14^	5473.0 (4782.0-6263.0)^c^
Optic pathway^d^	178 (11.1)	8.0 (0.1-56.8)	75 (99.8)	<0.001	7.0 (1.0-85.0)	445 (96.0);^15^	31 060.0 (25 907.0-37 237.0)^c^
High grade	28 (1.7)	25.9 (9.7-60.6)	8 (23.1)	0.04	58.0 (0-85.0)	77 454 (34.9);^16^	82.2 (56.6-119.5)^c^
Glioblastoma multiform^e^	18 (1.1)	25.2 (7.0-60.6)	4 (18.8)	0.01	64.0 (0-85.0)	33,951 (5.5)^16^	59.9 (37.6-95.3)^c^
Other^e^	10 (0.6)	30.2 (0.3-38.6)	4 (30.0)	NA	NA	NA	NA
Sarcoma^f^							
MPNST	243 (15.1)	33.3 (1.0-74.6)	72 (31.6)	0.003	46.0 (0-85.0)	2186 (43.4-71.9)^11^	9043.0 (7840.0-10 431.0)^c^
GIST	20 (1.2)	43.7 (24.9-68.6)	9 (80.0)	0.004	62.0 (18.0-101.0)	5138 (65.0-81.0)^12^	272.2 (175.0-423.4)^c^
ERMS	13 (0.8)	2.6 (1.0-61.4)	6 (63.6)	0.002	15.0 (0-85.0)	2831 (15.0-71.6);^11^	319.7 (185.0-552.4)^c^
UPS	5 (0.3)	36.8 (13.0-57.4)	1 (20.0)	0.01	57.0 (0-85.0)	14 599 (61.8-98.6)^11^	23.7 (9.9-57.1)^c^
Osteosarcoma	4 (0.2)	29.0 (17.4-44.0)	1 (50.0)	0.004	42.0 (0.1-78.8)	3482 (24.2-61.6)^13^	407.2 (152.2-1089.0)^c^
Breast carcinoma	47 (2.9)	44.2 (23.4-70.9)	27 (85.1)	0.78	62.0 (20.0-85.0)	3 597 331 (90.0)	3.8 (2.9-5.1)^c^
Endocrine neoplasia^g^							
Pheochromocytoma	20 (1.2)	44.9 (26.0-72.0)	8 (77.8)	0.01	47.1 (13.5-80.7)	107 (44.0-96.0)^19^	126.0 (81.0-195.9)^c^
Neuroendocrine tumor	9 (0.6)	56.6 (30.1-65.4)	7 (75.0)	0.04	63.0 (0-85.0)	35 618 (35.0-82.0)^20^	14.1 (7.3-21.1)^c^
Papillary thyroid carcinoma	7 (0.4)	49.4 (11.1-66.2)	4 (100)	0.17	51.0 (<20.0-85.0)	765 547 (98.0)	2.6 (1.2-5.4)
Skin cancer							
Melanoma	15 (0.9)	51.8 (34.3-82.5)	8 (66.7)	0.24	64.0 (<20.0-85.0)	1 245 276 (92.0)	3.9 (2.4-6.5)^c^
Nonmelanoma	14 (0.9)	68.6 (36.8-84.5)	4 (100)	NA	NA	NA	NA
Leukemia							
ALL	9 (0.6)	8.5 (2.1-38.3)	9 (100)	0.02	15.0 (<20.0-85.0)	100 012 (68.0)	28.2 (14.6-54.2)^c^
Other^h^	5 (0.3)	58.1 (3.8-73.8)	4 (100)	NA	NA	NA	NA
Genitourinary Neoplasia							
Ovarian serous carcinoma	8 (0.5)	48.8 (30.1-57.7)	4 (57.1)	0.09	63.0 (<20.0-85.0)	233 364 (47.0)	5.6 (2.8-11.1)^c^
Prostate adenocarcinoma	6 (0.4)	67.7 (31.8-77.9)	2 (100)	1.78	66.0 (35.0-85.0)	3 170 339 (98.0)	0.2 (0.1-0.5)^c^
Uterine adenocarcinoma	4 (0.2)	39.0 (31.6-54.6)	3 (100)	0.29	62.0 (20.0-85.0)	291 704 (81.0)	0.9 (0.3-2.3)
Lymphoma							
Hodgkin lymphoma	4 (0.2)	29.8 (23.2-44.2)	2 (100)	0.04	39.5 (<20.0-85.0)	215 531 (87.0)	6.2 (2.3-16.6)^c^
Non-Hodgkin lymphoma	2 (0.1)	48.9 (26.1-71.8)	2 (100)	0.16	67.0 (<20.0-85.0)	719 831 (71.0)	0.8 (0.2-3.1)
Other							
Meningioma	9 (0.6)	43.9 (27.3-57.8)	5 (100)	0.01	65.0 (7.0-87.0)	9000 (70.0)^21^	56.7 (29.4-109.1)^c^
Lung squamous cell carcinoma	6 (0.4)	68.8 (40.1-83.0)	2 (40.0)	0.13	71.0 (20.0-85.0)	248 102 (19.0)	2.9 (1.3-6.4)

Abbreviations: ALL, acute lymphoblastic leukemia; DSS, disease-specific survival; ERMS, embryonal rhabdomyosarcoma; GIST, gastrointestinal stromal tumor; MPNST, malignant peripheral nerve sheath tumor; NA, not applicable; NF1, neurofibromatosis type 1; OR, odds ratio; UPS, undifferentiated pleormorphic sarcoma.

^a^ Estimates were obtained from the Surveillance, Epidemiology, and End Results (SEER) Cancer Statistics Review 1975 to 2015 and SEER participants database unless otherwise specified (SEER sampled 34.6% of the US population in 2015).

^b^ Age at diagnosis comparisons are shown in eTable 3 in the Supplement.

^c^ In subgroup analysis, *P* < .001 considered statistically significant.

^d^ Unilateral or bilateral optic pathway glioma.

^e^ Includes 3 patients with gliosarcoma, 4 patients with anaplastic glioma, and 3 patients with unspecified high-grade glioma.

^f^ Not included: 2 patients with leiomyosarcoma, 2 patients with liposarcoma, and 1 patient with angiosarcoma.

^g^ Not included: 3 patients with pituitary adenoma, 1 patient with medullary thyroid carcinoma, and 1 patient with parathyroid adenoma.

^h^ Includes 2 patients with acute myelogenous leukemia, 2 patients with chronic myelogenous leukemia, and 1 patient with chronic lymphoblastic leukemia.

Deaths from disease were considered a DSS end point; other deaths were considered censored observations. Causes of death were determined based on the documentation of the electronic health record. The causes of death were reviewed by the research team composed of physicians (J.P.L., Y.C., and K.E.T.). We calculated DSS using the Kaplan-Meier method; survival curves were compared using the log-rank test. We measured DSS from diagnosis date to date of neoplasm-specific death or censorship. *P* values were 2-sided, and *P* < .05 was considered statistically significant. Owing to the concern for type I error from multiple secondary analyses, we adjusted our significance threshold to *P* < .001 for secondary analyses. Statistical analyses were performed using SPSS statistical software version 22.0 (IBM) from August 2018 to March 2020.

## Results

Among 2427 patients evaluated for NF1 during the study period, 1607 patients were included (Figure 1); 840 (52.3%) were female patients, and median (range) age at initial visit was 19 years (1 month to 83 years); 161 patients (10.0%) were younger than 8 years at last follow-up (Table 1). Of patients with NF1, 970 patients (60.4%) had no family history of neurofibromatosis; all patients had cutaneous, deep focal, or plexiform neurofibromas; 894 patients (55.6%) had documented deep focal or plexiform neurofibromas; and 271 patients (16.8%) had documentation of genetic testing at last follow-up.

The median (range) follow-up was 2.9 years (36.0 days to 30.5 years). In total, 666 patients (41.4%) developed nonneurofibroma neoplasms, of whom 550 patients (34.2%) developed a single neoplasm and 116 patients (7.2%) developed multiple neoplasms (Table 2). Patients with NF1, compared with the general population, developed several neoplasms at a younger mean (SD) age (low-grade glioma: 12.98 [11.09] years vs 37.76 [24.53] years; *P* < .0001; high-grade glioma [HGG]: 27.31 [15.59] years vs 58.42 [19.09] years; *P* < .0001; MPNST: 33.88 (14.80) years vs 47.06 (20.76) years; *P* < .0001; breast cancer: 46.61 [9.94] years vs 61.71 [13.85] years; *P* < .0001). At time of NF1 diagnosis, 53 patients (8.0%) had synchronous presentation of nonneurofibroma neoplasms. The study group had a 22.5% cumulative risk of neoplasia by age 30 years and 34.0% by age 50 years.

Patients with NF1 developed neoplasms more frequently compared with the general population (OR, 9.5; 95% CI, 8.5-10.5; *P* < .0001) (Table 2). The most common nonneurofibroma neoplasms by tumor type are displayed in Table 2. Among 889 neoplasms diagnosed in the study group, 682 were pathologically confirmed, while 207 neoplasms were confirmed by imaging only, including 175 optic pathway gliomas (OPGs), 31 non-OPG low-grade glioma (LGGs), and 1 HGG. In the study group, 551 patients (34.3%) had no brain imaging studies.

### Survival

**Figure 2.**
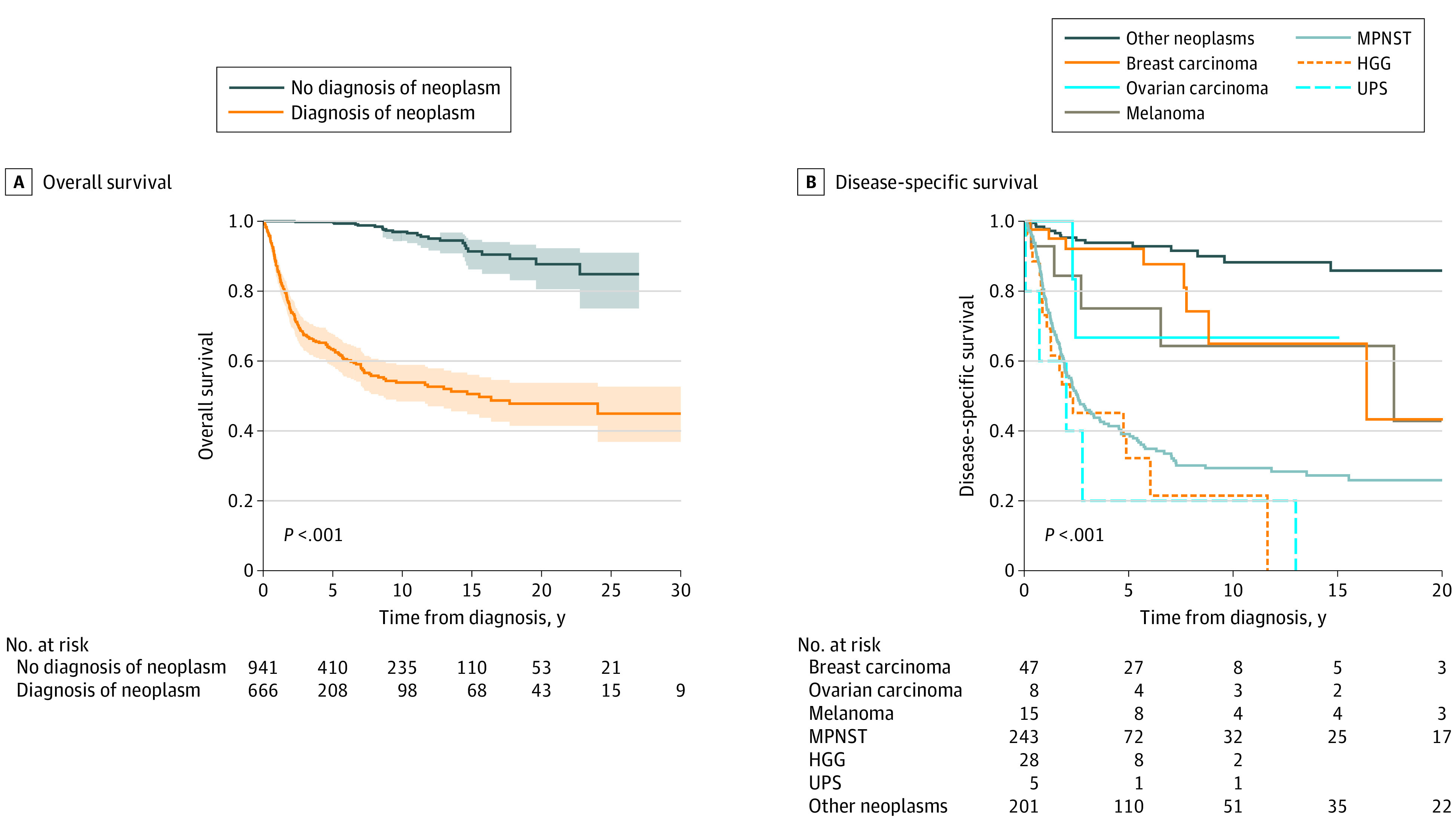
Kaplan-Meier Curves for Overall Survival and Disease-Specific Survival Panel B shows the most common histologies. HGG, indicates high-grade glioma; MPNST, malignant peripheral nerve sheath tumor; UPS, undifferentiated pleormorphic sarcoma.

The median (range) survival after diagnosis with a nonneurofibroma neoplasm for the NF1 group was 15.5 years (7.0 days to 46.6 years); Patients who were not diagnosed with a nonneurofibroma neoplasm had excellent outcomes, and we were not able to calculate median survival; the 25-year overall survival (OS) was 84.80% (95% CI, 75.00%- 94.60%). The survival curve did not cross 50% survival. The 5-year OS (SD) was significantly worse in patients with NF1 and nonneurofibroma neoplasms compared with patients without additional neoplasms (63.10% [0.05%] vs 99.60% [0.01%]; *P* < .0001) (Figure 2). Among 666 patients with NF1 who developed nonneurofibroma neoplasms, 261 patients (39.2%) died, with mean (SD; range) age at death 40 (18; 1-90) years. Among 941 patients with NF1 who did not develop additional neoplasms, 23 patients (2.4%) died, with mean (SD; range) age at death of 44 (19; 15-78) years. Among patients with NF1, significantly lower 5-year DSS rates were found among those with undifferentiated pleomorphic sarcomas (UPSs; 1 of 5 patients; [20.0%]), HGG (8 of 34 patients [23.1%]), MPNST (72 of 228 patients [31.6%]), ovarian carcinoma (4 of 7 patients [57.1%]), and melanoma (8 of 12 patients [66.7%]) compared with those who had neoplasms classified as *other* (110 of 119 patients [92.4%]) (all *P* < .001) (Table 2; Figure 2). No difference in DSS was found for other neoplasms when we compared the NF1 group with the general population.

Among patients with NF1 without nonneurofibroma neoplasms, 12 patients presented with more serious multisystemic complications that were associated with significant morbidity and mortality. These patients died at a younger mean (SD) age (29 [8] years); causes of death included complications from facial or airway neurofibromas for 3 patients, deep neurofibroma resection for 3 patients, neurological deficits from neurofibromas for 3 patients, unknown causes for 2 patients, and pneumonia for 1 patient.

### Glioma

Gliomas were the most common neoplasm in the NF1 group, found in 295 patients (18.4%). Within this subgroup, LGGs were the most frequently occurring, found in 267 patients (16.6%) (Table 2); OPG was the most prevalent subtype of LGG, found in 178 (11.1%) patients. Among patients with NF1 and a known OPG, 27 patients (15.2%) developed more than 1 LGG. Among patients with OPG, 56 patients (31.5%) received chemotherapy and 24 patients (13.5%) received additional radiotherapy (Table 3). Of 178 children with NF1 and radiographically identifiable OPGs, 68 (38.2%) developed signs or symptoms of the tumor. Among 89 patients with NF1 and non-OPG LGG, 33 patients (37.1%) received surgical resection. Twenty-eight patients developed HGG (1.7%), and among those, 6 patients (21.4%) developed HGG with a prior non-OPG LGG. The mean (SD) age at diagnosis of HGG for patients with NF1 was 27.31 (15.59) years vs 58.42 (19.09) years for the general population (OR 82; 95% CI, 56.6-119.5; *P* <.001; eTable 3 in the Supplement); 18 patients with HGG (64.3%) underwent surgical resection, of whom 16 patients (89.0%) received multimodality therapy (Table 3). Among patients who developed HGG, 9 patients received chemoradiation alone and 1 patient received chemotherapy only (eAppendix in the Supplement).

**Table 3.** Treatment for Most Frequent Nonneurofibroma Neoplasms in Patients With Neurofibromatosis Type 1

Neoplasm type	Treatment modality, No. (%)						
	Surgical treatment total	Surgical treatment alone	With chemotherapy	With radiation	Chemotherapy alone	Radiation alone	Chemoradiation alone
Glioma							
OPG (n = 178)	3 (1.7)	1 (0.6)	0	2 (1.1)	32 (18.0)	8 (4.5)	24 (13.5)
Non-OPG LGG (n = 89)	33 (37.1)	23 (25.8)	10 (11.2)	6 (6.7)	4 (4.5)	3 (3.4)	4 (4.5)
HGG (n = 28)	18 (64.3)	2 (7.1)	14 (50.0)	16 (57.1)	1 (3.6)	0	9 (32.1)
Sarcoma							
MPNST (n = 243)	223 (91.8)	36 (14.8)	155 (63.8)	128 (52.7)	14 (5.8)	2 (0.8)	2 (0.8)
GIST (n = 20)	18 (90.0)	6 (30.0)	7 (35.0)	0	0	0	1 (5.0)
ERMS (n = 13)	8 (61.5)	1 (7.7)	7 (53.8)	6 (46.2)	0	0	5 (38.4)
UPS (n = 5)	2 (40.0)	0	2 (40.0)	2 (40.0)	0	0	2 (40.0)
Breast carcinoma (n = 47)	46 (97.9)	0	44 (93.6)	38 (80.9)	0	0	1 (2.1)
Pheochromocytoma (n = 20)	20 (100)	0	2 (10.0)	1 (5.0)	0	0	0
Melanoma (n = 15)	14 (93.3)	5 (33.3)	6 (40.0)	7 (46.7)	0	0	0
NET (n = 9)	8 (88.9)	5 (55.6)	3 (33.3)	1 (11.1)	1 (11.1)	0	0
Ovarian carcinoma (n = 8)	8 (100)	0	8 (100)	2 (25.0)	0	0	0
Papillary thyroid carcinoma (n = 7)	7 (100	4 (57.1)	1 (14.3)	3 (42.9)	0	0	0

Abbrevations: ERMS, embryonal rhabdomyosarcoma; GIST, gastrointestinal stromal tumor; HGG, high-grade glioma; LGG, low-grade glioma; MPNST, malignant peripheral nerve sheath tumor; NET, neuroendocrine tumor; OPG, optic pathway glioma; UPS, undifferentiated pleormorphic sarcoma.

### Sarcoma

Among patients in the study group, 285 patients (17.7%) developed sarcomas. The most frequently occurring sarcoma subtype, MPNST, was diagnosed in 243 patients (15.1%). Of those, 5 patients (2.1%) developed 2 separate primary MPNSTs at the time of this study. All 248 MPNSTs developed from neurofibromas, and 79 patients with MPNST (32.9%) presented with metastatic disease at diagnosis (eTable 2 in the Supplement). Among patients with NF1, MPNSTs were diagnosed at a younger mean (SD) age compared with the general population (33.88 [14.80] years vs 47.06 [20.76] years; *P* < .0001) (eTable 3 in the Supplement). Among patients with MPNST, 223 patients (91.7%) underwent resection for MPNST; among these patients, 187 patients (83.9%) received neoadjuvant or adjuvant therapy (Table 3). Two patients with poor performance status at MPNST diagnosis received palliative care.

Among patients with NF1, 20 patients (1.2%) developed gastrointestinal stromal tumors (GISTs) and 13 patients (0.8%) developed embryonal rhabdomyosarcomas (ERMSs) (Table 2). Among these, 2 patients (10.0%) had metastatic GIST and 4 patients (30.8%) had metastatic ERMSs. Of the GISTs, none carried *c-kit* or *PDGFRA* mutations, 13 tumors (65.0%) were located in the jejunum or ileum, and 6 tumors (30.0%) were located in the duodenum. Among ERMSs, 11 sarcomas (84.6%) were located in genitourinary system. A diagnosis of ERMS was made 3-fold more often in male patients with NF1 (10 male patients and 3 female patients with NF1 developed ERMS) compared with 1.5 fold more often among male patients in sporadic cases.^23^ Other sarcomas in the NF1 group included UPS in 5 patients (0.3%), osteosarcoma in 4 patients (0.2%), leiomyosarcoma in 2 patients (0.1%), liposarcoma in 2 patients, and angiosarcoma in 1 patient (0.1%). Among patients with UPS, 2 patients (40.0%) had metastatic disease at diagnosis, whereas no patients had metastatic osteosarcoma, leiomyosarcoma, liposarcoma, or angiosarcoma at diagnosis; all patients diagnosed with UPS presented with a growing, painful mass and died from their disease. Among 4 patients with osteosarcoma, 2 patients (50.0%) showed now sign of progression after therapy and ultimately died of a synchronous primary MPNST.

### Breast and Ovarian Carcinoma

In the study group, 47 patients (2.9%), including 46 female patients and 1 male patient, developed breast cancer. Mean (SD) age at diagnosis for breast cancer in patients with NF1 was 46.61 (9.94) years compared with 61.71 (13.85) years in the general population (eTable 3 in the Supplement). Of women who developed breast cancer, 18 women (38.3%) developed it at younger than age 50 years, including 2 women (4.3%) who were diagnosed in their 20s. All breast cancers were ductal carcinoma; tumor receptor status is included in the eAppendix in the Supplement. Among patients with breast cancer, 3 patients (6.4%) had metastatic breast cancer at diagnosis (eTable 2 in the Supplement). Female patients with NF1 also developed ovarian carcinoma at a younger age and more frequently compared with general population estimates (OR, 5.6; 95% CI, 2.8-11.1; *P* < .0001) (Table 2). Among 8 patients with NF1 and ovarian serious carcinoma, 3 patients (37.5%) had ovarian carcinomatosis at diagnosis and 5 patients (62.5%) had developed peritoneal and distant metastasis by last follow-up.

### Pheochromocytoma and Neuroendocrine Tumors

In the study group, 36 patients (2.4%) developed endocrine neoplasms (Table 2); among these patients, 20 patients (1.2%) were diagnosed with pheochromocytoma. Of patients with NF1, 270 patients (16.8%) had hypertension by last follow-up. Of those, 20 patients (7.4%) had pheochromocytomas, among whom 2 patients (10.0%) were diagnosed with bilateral pheochromocytomas (eAppendix in the Supplement) and 2 other patients developed metastatic disease (eTable 2 in the Supplement). No difference in diagnosis age was found between the NF1 group and the general population. All patients with pheochromocytoma underwent surgical resection (Table 3). Among 666 patients who developed neoplasms, 9 patients developed neuroendocrine tumors (NET) (eTable 2 in the Supplement), among whom 8 patients (88.9%) underwent resection and 1 patient (11.1%) had metastatic NET at diagnosis. Among patients with pheochromocytoma, 1 patient was diagnosed with a rectal NET.

### Melanoma

Melanoma was diagnosed in 15 (0.9%) patients, among whom 6 patients (40.0%) had metastatic disease at diagnosis and 5 patients (33.3%) with localized disease at the time of surgery developed metastases postoperatively. The median (range) thickness of the primary melanomas was 2.7 (0.9-50.0) mm. Among patients with melanoma, 1 patient was diagnosed with periocular melanoma in the left orbit.

## Discussion

Individuals with NF1 can develop a wide variety of neoplasms, and the overall risk of neoplasm development among individuals with NF1 is 5% to 15% higher than in the general population, with an earlier age of onset and worse prognosis.^24^ This cohort study found that patients with NF1 had significantly lower DSS rates if they developed UPS, HGG, MPNST, ovarian carcinoma, or melanoma compared with other neoplasms. This study also found an increased prevalence of neoplasms among patients with NF1 (OR, 9.5; 95% CI, 8.5-10.5; *P* < .0001); however, this risk of neoplasm was primarily associated with tumors of the central nervous system and connective tissue.^6,24,25^ To our knowledge, this study is the largest single-center cohort study to date evaluating the characteristics of neoplasia in patients with NF1.

The *NF1* tumor suppressor gene encodes the neurofibromin protein, a negative regulator of the *Ras* oncogenic pathway.^26,27,28^ Inactivating mutations in *NF1* is associated with downstream activation of mitogen-activated protein kinase, phosphatidylinositol 3-kinase/protein kinase B/mechanistic target of rapamycin signaling, and uncontrolled cellular growth, differentiation, and survival, which is associated with the disease origin of NF1, including NF1-associated neoplasms.^2,26,27,28^ Few studies, to our knowledge, have examined the risk among individuals with NF1 of developing less common neoplasms or the outcomes associated with these neoplasms.^3^ In our study, 41.4% of patients with NF1 developed neoplasms other than neurofibromas. The most common neoplasms observed included LGG (16.6%), MPNST (15.1%), breast cancer (2.9%), HGG (1.7%), pheochromocytoma (1.2%), GIST (1.2%), and melanoma (0.9%).

The most common LGG diagnosed in patients with NF1 was OPG, found in 178 patients (11.1%); however, this rate was lower than a 2007 review^29^ estimate of 15% to 20%. This lower prevalence was likely associated with our observation that 551 patients (34.3%) in the NF1 group had no record of neuroimaging screening. Only half of children with NF1 and radiographically identifiable OPGs ultimately developed signs or symptoms of their tumor. Several non-OPG gliomas in our study group were detected and resected owing to symptoms. In addition, LGGs were significantly more frequent in our NF1 population than were sporadic LGGs in population estimates. A 2000 review^30^ found that NF1-associated LGGs undergo malignant transformation more frequently than comparable sporadic LGGs; thus, malignant transformation of NF1-associated LGGs may be associated with the increased prevalence and younger age of diagnosis for HGG among patients with NF1 in our study compared with the general population. Despite aggressive multidisciplinary treatment, HGG had a significant association with mortality in our study group. These findings suggest that conservative management should be used in patients with NF1 and asymptomatic LGGs, treatment should be recommended among patients with symptomatic or progressing lesions, and HGGs should be treated aggressively with a multidisciplinary approach.

Our study found that patients with NF1 were at significant risk of developing MPNSTs (OR, 9043; 95% CI, 7840-10 431; *P* < .0001) and that MPNST diagnosis occurred at a young age (mean, 33.88 years).^30^ The most common malignant neoplasm and cause of death in the NF1 group was MPNST; our findings agree with those from a 2012 study^31^ and a 2017 study^32^ that found a similar 5-year DSS among patients with NF1-associated MPNST. Often, MPNST-related symptoms overlap with those of growing neurofibromas, making diagnosis challenging. Larger tumors have been found to be indicators associated with a negative prognosis, suggesting the importance of early diagnosis and intervention for MPNST.^32^ These findings suggest that frequent evaluation should be performed among patients with NF1, especially those who have significant pain or rapid change in size of an existing neurofibroma.^33^

Although MPNST was the predominant sarcoma associated with NF1 in our study, non-neurogenic sarcomas were also more prevalent in the NF1 group than in the general population. In contrast to sporadic GISTs, of which most are found in the stomach, 65.0% of GISTs in our study group were located in the jejunum or ileum and 30.0% were found in the duodenum, consistent with the results of a 2005 study^34^ that found that these tumors were associated with the small bowel. Currently, there is no standard screening for GISTs; therefore, these findings suggest that careful attention should be given to patients with NF1 and possible GIST-related symptoms, such as recurring gastrointestinal pain, nausea and vomiting, positive fecal occult blood testing, or anemia.^35^

Another non-neurogenic sarcoma prevalent in our study group was ERMS. In contrast to sporadic ERMSs, most NF1-associated ERMSs in our study occurred in the genitourinary system (84.6%) and there was a higher proportion of cases in male patients (male to female ratio of 3:1 vs 1.5:1 in sporadic cases).^23^ In patients with NF1-associated ERMSs, the condition usually presents at an early age. Focused clinical exams with attention to common first symptoms (eg, scrotal mass, hematuria, or urinary retention) may be justified in boys with NF1.^10^

Studies from 1990 to 2013^36,37,38,39^ found an association between NF1 and rarer sarcomas in our study, but these studies are limited to case reports. In our study group, NF1-associated UPSs were aggressive despite multimodal therapy; all patients diagnosed with UPS died from their disease. These findings suggest that patients with NF1 should be frequently evaluated for symptomatic masses, given that all our patients with NF1 and UPS presented with a growing, painful mass. Although osteosarcomas are also rare in patients with NF1, 2 studies^38,39^ have found increased prevalence among these patients. Two patients in our study group had no evidence of osteosarcoma progression after therapy but ultimately died of a synchronous primary MPNST. Although rare, the association of leiomyosarcoma, liposarcoma, and angiosarcoma with NF1 may not be coincidental. Larger series are needed to further elucidate the association.^38,40,41,42,43,44^

A 2010 study^1^ found that individuals with NF1 have an increased risk of breast cancer. Our study results also found that young female patients with NF1 develop breast cancers frequently (OR, 3.8; 95% CI, 2.9-51; *P* < .0001), given that two-thirds of the breast cancers in our study group were diagnosed in women younger than age 50 years. These data suggest that recommendations should be followed from the National Comprehensive Cancer Network that screening mammography begin at age 30 in women with NF1.^45^ In addition, 2 patients in our study group were diagnosed with breast cancer in their 20s, which suggests the importance of annual clinical breast exams after age 18 years and consideration of magnetic resonance imaging for symptoms or palpable breast masses.

To our knowledge, no study to date has evaluated the risk and association of ovarian cancer in patients with NF1. Our study found an increased prevalence and decreased age at diagnosis of ovarian carcinoma among patients with NF1 compared with the general population. Most patients with NF1 and ovarian cancer had developed peritoneal and distant metastasis by last follow-up, which may be associated with the high mortality rate observed. Analysis of larger series is necessary to distinguish whether ovarian cancer screening would be associated with benefits among women with NF1.

An association between pheochromocytoma and NETs has been observed in patients with NF1; indeed, because of this association, a diagnosis of 1 condition should prompt an evaluation for the other.^46^ In our study, 1 patient with NF1 was diagnosed with both a pheochromocytoma and a rectal NET. Our findings suggest that these tumors should be considered in any patient with NF1 and NET-associated symptoms, such as gastrointestinal bleeding, flushing, obstruction, abdominal pain, obstructive jaundice, or pheochromocytoma.^46^

Compared with the general population, patients with NF1 in our study had significantly higher rates of pheochromocytoma (OR, 126; 95% CI, 81-195; *P* < .0001) and NET (OR, 14.1; 95% CI, 7.3-21.1; *P* < .0001). About 0.2% of the general population with hypertension develops sporadic pheochromocytomas.^47^ Patients with NF1 frequently have essential hypertension or vasculopathy, making diagnosis of pheochromocytoma difficult.^48^ Given the sensitivity of biochemical tests for catecholamines combined with abdominal imaging, patients with NF1 and refractory hypertension or symptoms of catecholamine excess should be evaluated for pheochromocytoma, given that this disease can metastasize.^48^ Definitive treatment is surgical treatment; however, about 16% of patients experience disease recurrence despite successful resection.^49^ Thus, long-term monitoring is indicated for all patients with NF1, even after successful resection.^49^

Two studies^50,51^ found an association between NF1 and melanoma, and our study found a significantly increased risk for melanoma among patients with NF1 (OR, 3.9; 95% CI, 2.4-6.5; *P* < .0001). Of our patients with NF1 and melanoma, 40.0% presented with metastatic disease at diagnosis, which is a higher proportion than in the general population (ie, 4%).^52^ Furthermore, survival was worse in the NF1 group compared with the general population. Melanomas associated with NF1 melanomas had a median thickness of 2.7 mm, which supports previous findings that NF1-associated melanomas are thicker than sporadic melanomas (median thickness, 1.5 mm).^51^ Our findings suggest the importance of maintaining a high index of suspicion for melanoma in patients with NF1 and performing frequent, meticulous skin and ocular exams.

### Limitations

 This cohort study has some limitations. First, some patients with NF1 may have received treatment elsewhere, although this would suggest a higher prevalence of neoplasia than that found in our analysis. Second, the patients lost to follow-up may be associated with changes in the outcome analyses. Third, 10.0% of our NF1 group was younger than age 8 years old at last follow-up, which may be associated with changes in rates of clinical characteristics and prevalence or risk of neoplasia in our group; these younger patients with NF1 may be associated with decreases in the true numbers of characteristics and patients at risk for non-NF neoplasia. Fourth, the mean age for patients with NF1 who did not develop additional neoplasms was similar to the mean age for those who did develop neoplasms. This may be associated with the small sample size. Despite these limitations, our study found neoplasia occurrence and patient outcome characteristics that may be used in the evaluation of neoplasia among patients with NF1.

## Conclusions

This cohort study of 1607 patients found that patients with NF1 had significantly lower DSS rates if they developed UPS, HGG, MPNST, ovarian carcinoma, or melanoma compared with other neoplasms. Individuals with NF1 were found to have significantly increased rates of multiple neoplasms other than neurofibromas. Some of these are classically associated with NF1, and others were previously not associated with NF1. These neoplasms had significant associations patient outcomes. As larger studies of NF1-associated cancers emerge, understanding of the neoplasia drivers and mechanisms associated with outcomes among individuals with NF1 may improve. This study’s results may inform counseling of patients with NF1 and support a multidisciplinary approach to care.

## Notes

Supplement. **eTable 1.** Clinical Diagnostic Criteria for Neurofibromatosis Type 1 **eTable 2.** Presentation and Outcomes for the Most Frequently Occurring Neoplasms in Patients With Neurofibromatosis Type 1 **eTable 3.** Mean Age at Cancer Diagnosis for Patients With and Without Neurofibromatosis Type 1 **eAppendix.** Neurofibromatosis Type 1 Neoplasia Characteristics and Locations
