# Supplementary material for: Comparison of Cancer Prevalence in Patients With Neurofibromatosis Type 1 at an Academic Cancer Center vs in the General Population From 1985 to 2020
Source: JAMA Netw Open. 2021 Mar 18;4(3):e210945. doi: 10.1001/jamanetworkopen.2021.0945 (PMC7974640; doi:10.1001/jamanetworkopen.2021.0945)
Supplement: Supplement. — eTable 1. Clinical Diagnostic Criteria for Neurofibromatosis Type 1 eTable 2. Presentation and Outcomes for the Most Frequently Occurring Neoplasms in Patients With Neurofibromatosis Type 1 eTable 3. Mean Age at Cancer Diagnosis for Patients With and Without Neurofibromatosis Type 1 eAppendix. Neurofibromatosis Type 1 Neoplasia Characteristics and Locations [file jamanetwopen-e210945-s001.pdf]

Supplemental Online Content

Landry JP, Schertz KL, Chiang YJ, et al. Comparison of cancer prevalence in patients with neurofibromatosis type 1 at an academic cancer center vs in the general population from 1985 to 2020. *JAMA Netw Open*. 2021;4(3):e210945. doi:10.1001/jamanetworkopen.2021.0945

eTable 1. Clinical Diagnostic Criteria for Neurofibromatosis Type 1

eTable 2. Presentation and Outcomes for the Most Frequently Occurring Neoplasms in Patients With Neurofibromatosis Type 1

eTable 3. Mean Age at Cancer Diagnosis for Patients With and Without Neurofibromatosis Type 1

eAppendix. Neurofibromatosis Type 1 Neoplasia Characteristics and Locations

This supplemental material has been provided by the authors to give readers additional information about their work.

© 2021 Landry JP et al. *JAMA Network Open.*

eTable 1. Clinical Diagnostic Criteria for Neurofibromatosis Type 1[7]
**The presence of 2 or more of the following criteria are diagnostic of NF1**
-6 or more café-au-lait macules > 5 mm in diameter in prepubertal or > 15 mm in postpubertal individuals
-2 or more neurofibromas of any type or 1 plexiform neurofibroma
-Axillary and/or inguinal freckling
-Optic nerve glioma
-2 or more small elevated iris hamartomas (Lisch nodules)
-A distinctive osseous lesion, such as sphenoid wing dysplasia or long bone cortex thinning with or without pseudoarthrosis
-A first-degree relative with NF1 according to the above criteria

Abbreviation: NF1, neurofibromatosis type 1.

© 2021 Landry JP et al. *JAMA Network Open.*

eTable 2. Presentation and Outcomes for the Most Frequently Occurring Neoplasms in Patients With Neurofibromatosis Type 1

**Malignancy Type**	**Total (N)**	**Metastasis at diagnosis (%)**	**NED (%)**	**Median survival (years)**	**Median time to death from diagnosis, year (range)**
MPNST	243	33	25	2.3	1.5 (0.1-15.5)
Breast carcinoma	47	6	49	16.4	5.8 (1.1-16.4)
High-grade glioma	28	7	7	1.8	1.6 (0.1-11.6)
Pheochromocytoma	20	5	88	n/a	2 (1.2-2.9)
GIST	20	10	45	n/a	1.9 (1.4-2.5)
Melanoma	15	40	27	5.7	4.0 (0.3-17.7)
ERMS	13	31	38	8.3	1.1 (0.5-8.3)
ALL	9	n/a	100	n/a	-
NET	9	11	33	14.7	1.2 (0.5-14.7)
Ovarian carcinoma	8	38	38	11.6	2.3 (1.2-11.6)
Papillary thyroid carcinoma	7	14	86	n/a	-
Prostate adenocarcinoma	6	0	50	n/a	5.16
Lung SqCC	6	67	0	1.0	0.7 (0.3-1.2)
UPS	5	40	0	2.0	2 (0.1-13)
Osteosarcoma	4	0	25	7.3	1.1

Abbreviations: MPNST, malignant peripheral nerve sheath tumor; GIST, gastrointestinal stromal tumor; ERMS, embryonal rhabdomyosarcoma; UPS, undifferentiated pleomorphic sarcoma; ALL, acute lymphoblastic leukemia; n/a, not applicable; NET, neuroendocrine tumor; Lung SqCC, lung squamous cell carcinoma; NED, no evidence of disease at last follow-up; NF1, neurofibromatosis type 1; DSS, disease-specific survival by end of study

© 2021 Landry JP et al. *JAMA Network Open.*

**eTable 3.** Mean Age at Cancer Diagnosis for Patients With and Without Neurofibromatosis Type 1							
**Malignancy Type**	**Number of NF1 patients (N)**	**Mean age at diagnosis for NF1 patients (years)**	**Standard deviation mean age (years)**	**Number of patients general population[a](N)**	**Mean age at diagnosis for general population (years)**	**Standard deviation mean age (years)**	***p* value**
Low-grade gliomas	267	12.98	11.09	10214	37.76	24.53	<0.0001[b]
High-grade gliomas	28	27.31	15.59	36827	58.42	19.09	<0.0001[c]
MPNST	243	33.88	14.80	1032	47.06	20.76	<0.0001[b]
Breast cancer	47	46.61	9.94	616669	61.71	13.85	<0.0001[b]

[a]The data was extracte d from SEER database (1985-2017).[b]Two-sample Wilcoxon rank-sum test; [c]T test
Subgroup analysis: P-value considered statistically significant as p<0.001

© 2021 Landry JP et al. *JAMA Network Open.*

eAppendix. Neurofibromatosis Type 1 Neoplasia Characteristics and Locations

Glioma (n=295 neoplasms)

Non-optic pathway low grade gliomas were located in many regions of the nervous system: the brainstem (n = 29); cerebrum (n = 29); cerebellum (n = 22); thalamus (n = 6); and hypothalamus (n = 3). High grade gliomas were located in the cerebrum (n = 15), cerebellum (n = 8), and brainstem (n = 5). Patients managed with chemoradiation alone had either diffuse infiltrating brain HGG (n=4). One patient received chemotherapy alone and is currently undergoing treatment due to recent diagnosis before end of study.

Sarcoma (n=295 neoplasms)

Locations of the 248 MPNSTs were in the lumbosacral nerve (n=61), brachial plexus (n=39), tibial nerve (n=21), thoracic nerve (n=20), lower extremity sciatic nerve (n=16), cervical nerve (n=14), femoral nerve (n=12), truncal sciatic nerve (n=12), cranial nerve (n=10), median nerve (n=6), iliac nerve (n=4), ulnar nerve (n=3), radial nerve (n=2), and the phrenic nerve (n=1). The exact nerve source was unknown in 27 of the MPNSTs, but they were located in the mediastinum (n=9), head (n=5), intra-abdominal (n=4), proximal upper extremity (n=4), proximal upper extremity (n=3), foot (n=1), and hand (n=1). GIST locations were jejunal/ileal in 13 patients, duodenal in six, and gastric in one. The median GIST size was 5 cm (range, 0.3 cm – 21 cm) and median mitotic rate was 2/50 high-power fields (HPF) (range, 0-19). ERMS were located in the genitourinary tract in 11 patients and the head in two. UPS were located in the back, mediastinum, thigh, upper extremity, and shoulder. Osteosarcomas were located in the leg in two patients, the chest in one, and the thigh in one. Leiomyosarcomas were located in the abdomen and the thigh. Both liposarcomas were located in the thigh. One angiosarcoma was located at the femoral head.

Breast Cancer (n = 47)

© 2021 Landry JP et al. *JAMA Network Open.*

All breast cancers were ductal carcinoma; 29 (61.7%) tumors were estrogen receptor positive, 22 (46.8%) were progesterone receptor positive, and 12 (25.5%) were HER2/neu positive. Four (8.5%) patients had triple-negative breast cancer. Receptor status was unknown in six (12.8%) patients.

Pheochromocytoma and Neuroendocrine Tumors (n=29)

Primary pheochromoctyomas were right-sided in nine patients, left-sided in nine patients, and bilateral in two patients. Primary neuroendocrine tumors were located in the pancreas in three patients, small bowel in two, colon in two, lung in one, and ovary in one.

© 2021 Landry JP et al. *JAMA Network Open.*
